# Contagious Bovine Pleuropneumonia: Seroprevalence and Risk Factors in Gimbo District, Southwest Ethiopia

**DOI:** 10.1155/2018/5729296

**Published:** 2018-05-24

**Authors:** Yosef Mamo, Molalegne Bitew, Tsegaye Teklemariam, Murga Soma, Debebe Gebre, Temesgen Abera, Tefera Benti, Yosef Deneke

**Affiliations:** ^1^Jimma University, College of Agriculture and Veterinary Medicine, Jimma, Ethiopia; ^2^Ethiopian Biotechnology Institute, P.O. Box 32853, Addis Ababa, Ethiopia; ^3^National Animal Health Diagnostic and Investigation Center (NAHDIC), Sebeta, Ethiopia

## Abstract

Contagious bovine pleuropneumonia (CBPP) is a highly contagious disease of cattle which is one of the great plagues which continues to devastate the cattle herds on which so many people are dependent in Africa. Cross-sectional study was conducted from October 2015 to August 2016 to determine the seroprevalence of CBPP in cattle and associated risk factors in Gimbo district, Southwest Ethiopia. A total of 384 serum samples were collected and tested for the presence of specific antibodies against Mycoplasma mycoides subspecies mycoides small colony (MmmSC), using a competitive enzyme-linked immunosorbent assay (cELISA). Univariable and multivariable logistic regression analysis were performed to determine the association between risk factors and seroprevalence of CBPP. An overall seroprevalence of CBPP was 8.1% (31/384) and it was ranging from 0% to 20% across different Peasant associations (PAs). The seroprevalence of CBPP among adult animals was 8.5% (25) and in young 6.6% (6), in good body condition animals 6.6% (18) and in poor 11.5% (13), in dry season 11.9% (20) and in rainy 5.1% (11), and in highland altitude 2.5% (3), midland 3.8% (5), and lowland 17.4% (23). Among the potential predisposing factors assessed, altitude was found significantly (p = 0.02, OR = 7.3) associated with the seroprevalence of contagious bovine pleuropneumonia and other risk factors had no significant (P > 0.05) influence. The present study showed that the overall seroprevalence of CBPP in Gimbo district was high and this indicates a need for intervening and implementing control measures to prevent further spread of the disease in the district through the use of better and coordinated vaccination program.

## 1. Introduction

Ethiopia is one of the countries in African continent with huge livestock potential and among the first ten in the world [1]. It owns a vast number of livestock with an estimated 56.71 million cattle, 29.11 million goats, and 29.33 million sheep [2]. However, the productivity of this sector is lower than the African production average [3]. The output per unit of domestic breed of livestock is very low due to the fact that the animal husbandry methods are traditional [4].

Low genetic potential of the animals, poor nutrition, and widespread diseases are the major constraints contributing to the low productivity of local breeds [5, 6]. Transboundary animal diseases such as contagious bovine pleuropneumonia (CBPP) cause the major limitation to the livestock agriculture of the country and affect livelihood through their effect on animal health and impact on the production, availability, and quality of animal food [4].

Contagious bovine pleuropneumonia (CBPP) is caused by* Mycoplasma mycoides* subspecies* mycoides *small colony (*Mmm*SC) which is a highly contagious respiratory disease of cattle found in most sub-Saharan African countries [7, 8]. CBPP has been considered the most serious disease of cattle after the global eradication of rinderpest was declared in 2011 [8, 9]. CBPP is now recognized as a priority trans-boundary animal disease on the basis of its transmissibility and economic impacts [8].

CBPP is endemic to part of Africa, although it has been eradicated in other parts of the world through the application of restrictions to the movement of cattle, as well as test and slaughter policies combined with compensation for livestock owners during the mid 20^th^ century [10]. The aforementioned policies are difficult to apply in most African countries and the disease is rampant in pastoral areas of Africa and is a major setback for Ethiopian livestock development. The incidence of the disease began to decline in Africa by the 1970s. However, as a consequence of the economic and financial difficulties that affected the capacity of governments to adequately fund veterinary services, the disease came back in the late 1980s and early 1990s [11, 12]. Consequently, the disease remains endemic in Africa particularly in tropical and subtropical regions (west, central, east, and parts of southern Africa) of the continent [7, 13, 14].

According to Abdella and Yune [8], outbreaks were reported in 20 African countries like Ghana, Burkina Faso, Senegal, Congo, and so on. In Mali the national prevalence of 18.11% at the individual level and 85.93% at the herd level has been reported [15]. The highest number of cases was recorded in Ethiopia in the last few years [8]. The occurrence of such diseases impacts both extensive and intensive livestock producers by marginalizing them from higher price livestock markets and restricting their capacity for value-added trade [16].

CBPP has been reported in different regional states of Ethiopia with an overall seroprevalence like 7.13% in Afar, 1.29% in Amhara, 12.05% in Benishangul Gumuz, 19.72% in Gambella, 5.17% in Oromia, 5.44% in Southern Nations Nationalities and People (SNNP), 0.9% in Somali, and 6.11% in Tigray in the year of 2004 [17]. The main risk factors of CBPP infection were found to be altitude and herd size [18].

To accomplish an effective control of CBPP through strategic vaccination, among the prerequisites are thorough understanding of the epidemiology of the disease in the country. Hence, from the outset the epidemiological assessment of CBPP should be implemented in order to envisage a rational plan for the control and eventual eradication of CBPP from Ethiopia [8]. However, there is no published report on the prevalence and associated risk factor of CBPP in Gimbo district except Abdela and Yune [8] who reviewed the output of this study project and only limited information is available about the epidemiological status of the CBPP in the study area. Besides, there were suspected reports of CBPP outbreak in the study area by field veterinarian. This was the pertinent rationale to initiate this study project. Hence, this study was undertaken with the objective to determine the animal level seroprevalence and associated risk factors of contagious bovine pleuropneumonia in Gimbo district of Southern Nations, Nationalities and People's Regional State (SNNPRS).

## 2. Materials and Methods

### 2.1. Description of the Study Area

**Figure 1:**
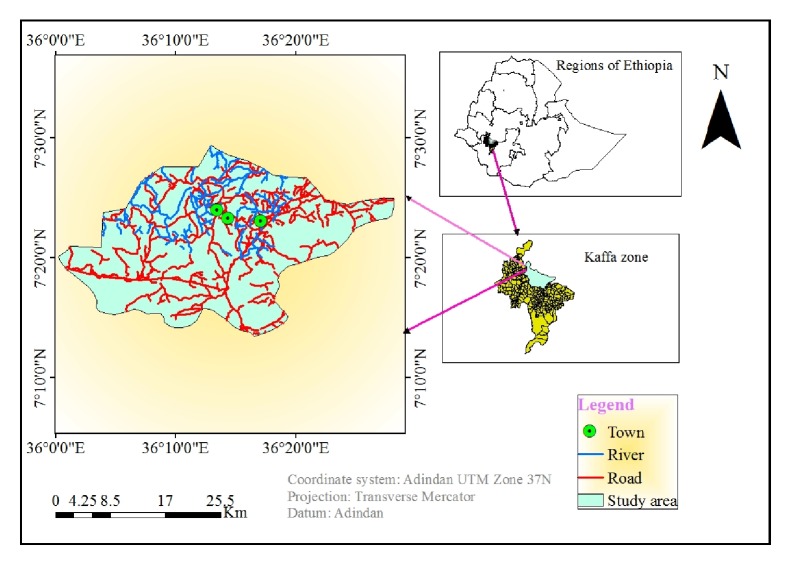
Map of the study area.

The present study was conducted from October 2015 to August 2016 in Gimbo district, Keffa zone, Southern Nations, Nationalities and People's Regional State, Southwest Ethiopia. The district is 385 km far away from Addis Ababa, capital city of Ethiopia. Geographically, it is located at 07° 24' 55.2” N and 36° 13' 60”E and its altitude ranges from 1050 to 3500 meters above the sea level. The total area estimated is at about 871.86 square kilometers and it is bound at North with Gojeb River, West by chena district, East by Menjiwo district, and South by Decha district. The district has 31 Peasant associations (PAs) of which 15.3%, 63.4%, and 21.3% account for highland, midland, and lowland, respectively. An average annual temperature and rain fall ranges from 20°C to 31°C and 900 to1150 mm, respectively (Figure 1).

### 2.2. Study Animal

The study population comprised 104,901 cattle of above six months of ages managed under the traditional extensive production system and all study animals from which blood samples were collected were apparently healthy and with no history of vaccinations to CBPP.

### 2.3. Study Design

The cross-sectional type of study was designed to determine the seroprevalence and associated risk factors of CBPP in Gimbo district.

### 2.4. Sample Size Determination

Sample size was determined according to Thrusfield [19] formula considering 50% expected prevalence since there was no previous study specifically in the study area and due to the limitation of published research in neighboring zone with similar agroecological area.

(1) $$\begin{array}{c} N=\frac{1.96^{2}Pexp\left(1-Pexp\right)}{d^{2}}=1.96^{2}\frac{0.5\left(1-0.5\right)}{.05^{2}} \end{array}$$

where, n is sample size of the study population, d is absolute desired precision (0.05) (±5%, i.e., the limits of the associated 95% interval), p is previous/expected prevalence in the study area, and CI is confidence interval. Based on the above formula, 384 animals were selected.

### 2.5. Sampling Methods

The total of 31 PAs were categorized into three agroecological categories as highland, midland, and lowland; then, from each agroecology, three PAs were selected conveniently based on accessibility, availability of infrastructure, and number of cattle population. The households having cattle were selected conveniently from nine PAs based on accessibility of infrastructure and number of cattle population. The selected households were informed by data collectors to provide their cattle for sampling purpose. Animals from each household were tethered separately by object. The number was assigned to animal by counting tethered animals either from right to left or from left to right. Simple random sampling techniques were used to select animal for serum sample collection.

### 2.6. Sample Collection

7-10 ml of blood samples was collected aseptically from the jugular vein using plain vacutainer tubes. The samples were kept under the shade at an angle of 45° in a slant position for four up to six hours to allow clotting of blood and serum separation. The serum samples were transferred to cryovial, labeled, stored in ice box and transported to Mizan regional laboratory, and kept at -20°C until processing [1]. The stored serum was transported to National Animal Health Diagnostic and Investigation Center (NAHDIC). Corresponding to each sample the sample code, age, sex, body condition, agroecology of the area, PAS, season, and georeference information were collected and registered on vacutainer tube and a separate case book. Age determination and body condition scoring were done according to the standard protocol.

### 2.7. Competitive Enzyme-Linked Immunosorbent Assay (cELISA)

Competitive enzyme-linked immunosorbent assay (cELISA) was used as recommended by CIRAD-UMR15 (France) and is based on a monoclonal anti-*Mmm*SC antibody named Mab 177/5 (OIE, 2014). Microplates were coated with* Mmm*SC purified lysate. Samples to be tested were premixed with the specific monoclonal antibody Mab117/5 in a separate plate (“preplate”) and content of the preplate is transferred in to the coated microplate. Any* Mmm*SC specific antibodies present in the sample will form an immune complex with* Mmm*SC antigen coated on the microplate competing with Mab117/5 for the specific epitope. After washing away unbounded material, an anti-mouse antibody enzyme conjugate was added. In presence of immune complex between* Mmm*SC antigen and antibodies from the sample, Mab117/5 cannot bind to its specific epitope and the conjugate is blocked from binding to Mab117/5. Conversely in the absence of* Mmm*SC antibodies in the test sample, MAb117/5 can bind to its specific epitope and the conjugate is free to bind to MAb 117/5. Unbound conjugate was washed away and enzyme substrate Tetra methyl Benzedrine (TMB) was added. In presence of enzyme, the substrate is oxidized and develops a blue color becoming yellow after adding stop solution. Subsequent color development inversely proportional to the amount of anti-*Mmm*SC antibodies in the test sample. The underside of plate was wiped and optical density (OD) of individual reactions was measured at 450 nm using a plate reader. The percentage inhibition (PI) value for each sample was calculated by the following formula.

(2) $$\begin{array}{c} PI=\frac{\left(OD\;\;Mab-OD\;\;test\;\;serum\right)x100\%}{OD\;\;Mab-OD\;\;conjugate} \end{array}$$

where OD Mab is optical density for the monoclonal antibody; OD test serum is optical density for the test serum; OD conjugate is optical density for the conjugate [1].

### 2.8. Interpretation of Result

Samples with percentage of inhibition less than or equal to 40% are considered as negative for the presence of* Mmm*SC antibodies. Samples with the percentage of inhibition greater than 40% and less than 50% are considered doubtful, whereas samples with percentage of inhibition greater than or equal to 50% are considered positive for presence of* Mmm*SC antibodies. The specificity of cELISA was 99.9% and sensitivity was 63.8% [1].

### 2.9. Data Analysis

All data were coded and stored in Microsoft excel spread sheet and screened for proper coding and any errors. It was transferred to SPSS version 20 and analysis was performed. The overall seroprevalence was estimated by dividing the number of cELISA test positive animals by the total number of animals tested. The statistical significance difference seroprevalence based on difference in risk factors was tested by chi-square (X^2^) test. Univariable and multivariable logistic regression were used to determine the association between explanatory variables (risk factors) and the dependent variable (serological status of the animals). In all analysis confidence level and absolute precision were 95% and 5%, respectively, and* p *< 0.05 was set for significance.

## 3. Results

Out of 384 cattle serum examined, 8.1% (31/384) were positive for contagious bovine pleuropneumonia specific antibody. The highest seroprevalence was observed in Choba kebele 20% (10), whilst the lowest seroprevalence was recorded in Shocha (0%). The study found that seroprevalence of CBPP was significantly (*p *< 0.05) associated with PAs (Table 1).

Out of 216 cattle examined during the rainy season (March and April), 5.1% (11) were found positive for CBPP and out of 168 cattle examined during the dry season (January and February), 11.9% (20) were found positive for CBPP (Table 2).

The effects of age, sex, body condition, altitude, and season on the seroprevalence of CBPP were analyzed by univariable logistic regression. It revealed that altitude and season had significant (p < 0.05) association with seroprevalence of CBPP (Table 3).

Multivariable analysis of risk factors and their impact on the seroprevalence of CBPP found that agroecology was significantly (*p *< 0.05) associated with seroprevalence of CBPP whereas season had no significant (*p *> 0.05) association with seroprevalence of CBPP. The odd of CBPP seropositivity was 7.3 times higher in animals taken from lowland compared to highland areas (Table 4).

## 4. Discussion

The present study was designed to determine seroprevalence and associated risk factors of contagious bovine pleuropneumonia in cattle in Gimbo district of southwest Ethiopia and the results clearly showed that the disease is rampant in the study area. Animals that tested positive for antibodies to* M. mycoides subspecies mycoides* small colony are habitual carriers of the disease without any clinical signs but are potential source of infection to other susceptible animals.

In this study, an overall seroprevalence of 8.1% (31/384) was detected in cattle by cELISA. This finding was in line with the results of different other studies conducted in different parts of Ethiopia reported as 8.7% in Bishoftu using cELISA [20], 9.4% in Borena using cELISA test [21], 9.5% in Export quarantine center Adama [22], 10% in Dassenech district of South Omo zone [23], and 11.9% in Southern part of Tigray testing using CFT test [24]. Similar seroprevalence results are also reported from other countries comparable with the present study like 9.7% in Southwestern Kenya [25] and 10.65% in Kwara state, Nigeria [26]. However, the overall seroprevalence is lower than other reports like 39% reported by [18] in Somali Regional State and 28.5% reported by Daniel et al. [27] in three districts (Gobbu Sayyo, BakoTibbe, and Horro) of Western Oromia, Ethiopia. Still there are also reports which are less than the present report like 0.4 % reported by Gezahegn et al. [28] in Borena and 0.4% reported by Erimiyas et al. [29] in Export quarantine center of Adama. The variation of this finding in seroprevalence reported from different parts of Ethiopia and other countries might be due to variation in temporal and spatial distribution of the disease, differences in agroecological systems, cattle management and production systems, population density, number of examined animals, and the types of tests used to determine the seroprevalence.

Among the potential predisposing factors assessed age (young 6.6% and adult 8.5), sex (male 7.1% and female 8.8%), and body condition (good 6.6% and poor 11.5%) had no significant (*p *> 0.05) association with seroprevalence of CBPP. Seropositivity in adults was a bit higher than that in young animals. This small difference in seroprevalence between the two age categories could be attributed to the fact that young animals are usually tethered within the homestead when adult cattle go to graze. This is to save them from the long exhaustion suffered by older animals that cover long distances in search of good pasture and water where they interact with herds from other households. With this kind of practice, young animals do not normally have direct contact with other herds other than that of the dams. Therefore; there is less chance to come into contact with infected animals.

The present finding also corroborated with Teshale et al. [24] who reported that age and sex were not significantly (*p *> 0.05) associated with seroprevalence of CBPP. Daniel et al. [27] reported that age, sex, and body conditions were not significantly (*p *> 0.05) associated with seroprevalence of CBPP and Biruhtesfa et al. [20] also reported that age was not significantly associated with occurrence of CBPP. The insignificant difference of seroprevalence among the age groups, sexes, and body conditions might be due to similar exposure of animals to the disease: since the disease is contagious all animals in the herd could be infected due to the chronic nature of the disease. The disease is mainly transmitted from animal to animal through aerosol. This organism can be found in saliva, urine, fetal membranes, and uterine discharges [30]. This could play great role in uniformity of infection in all age groups, sexes, and body conditions.

This finding contradicts the finding of Bashiruddin* et al.* [31] who reported that with age variation infection resistance could also vary. According to Bashiruddin* et al.* [31] animals less than 3 years of age are less resistant to experimental challenge. In two separate experiments, it was shown that cattle over 3 years of age were more resistant to CBPP infection than younger animals. In addition, Masiga et al. [32] reported that young animals are more susceptible to acute forms of CBPP infection than adult cattle and thus acutely infected young animals may die of CBPP and may not be available for testing. However, the study made by Kassaye and Molla [22] and Andrew et al. [33] contradicts Bashiruddin* et al.* [31] who both stated that calves were less positive to CBPP seroprevalence.

Among the potential predisposing factors assessed, agroecology (lowland) was significantly (*p *< 0.05) associated with the seroprevalence of the disease. This result was in line with Teshale et al. [24] in Southern Zone of Tigray Regions and Gedlu [18] in Somali region who reported significant difference of seroprevalence of CBPP with difference in the agroecology. This might be due to the fact that in lowland area there is lack of good pasture and water and presence of more concurrent disease could predispose to CBPP infection. Factors such as extremes of age, stress, and concurrent infections may predispose to tissue invasion [34]. Also in lowland areas animals move long distance in search of pasture and water and they interact with other infected herds. In addition to this, lowland animals are more confined to grazing area and watering point so CBPP could be easily transmitted through aerosol to susceptible animals. Transmission is favored by close crowding of cattle, and outbreaks are more common and extensive when cattle are housed or have been transported by train or truck or trekked on foot in groups [1]. The environment may weaken the host and increase its susceptibility to infection or provide conditions that favor the survival of the agent [19].

Higher prevalence was recorded during the dry season even though statistical analysis revealed that there is no significant (*p *> 0.05) association between season and seroprevalence of CBPP. This result contradicts that of Gezahegn et al. [28] who reported that there is significant association between season and seroprevalence of CBPP. Insignificant association between the season and seroprevalence might be due to the fact that animals are free moving (not housed) and they come in close contact of each other during both the rainy and dry season equally and this is the time when CBPP infection is common.

In general, epidemiological evidence of the present investigation revealed that CBPP was considerably prevalent disease in the study area and needs due attention from farmers and veterinarians as well as from government for successful adoption of policy to control and eradicate the disease from the country especially in the lowland area.

## Figures and Tables

**Table 1:** Seroprevalence of CBPP based on altitude tested by *X*^*2*^.

Agroecology	PA	No. tested	No. positive	Seroprevalence (%)	95% CI	*X* ^*2 *^(*p* value)
Highland	Kuti	45	2	4.4	0.04-1.07	3.34 (0.037)
	Dakit	40	1	2.5	0.04-6.3	
	Shocha	37	0	0	-	
Midland	Amani	43	1	2.3	0.04-5.8	3.34 (0.037)
	Michit	41	2	4.9	0.14-8.2	
	Afudo	46	2	4.3	0.13-7.2	
Lowland	Shombakichib	37	5	13	0.6-18.4	
	Choba	50	10	20	1.1-26	3.34 (0.037)
	Boka Welega	45	8	17.7	0.92-23.2	
	Total	384	31	8.1	5.3-10.8	

**Table 2:** Effect of risk factors on seroprevalence of CBPP tested by *X*^*2*^ test.

Variable	No. tested	No. positive	Seroprevalence (%)	*p* value	95% CI
Age					
Young (6m-2yrs)	91	6	6.6	0.554	0.03-1.9
Adult (>2yrs)	293	25	8.5		0.52-3.3

Sex					
Male	169	12	7.1	0.536	0.37-1.6
Female	215	19	8.8		0.59-2.6

Body condition					
Good	171	18	6.6	0.115	0.29-1.15
Poor	113	13	11.5		0.8-3.8

Season					
Rainy	216	11	5.1	0.018	0.18-0.8
Dry	168	20	11.9		1.17-5.4

Altitude					
Highland	122	122	2.5	0.01	0.03-0.4
Midland	130	130	3.8		0.37-6.7
Lowland	132	132	17.4		1.17-5.4

**Table 3:** Univariable logistic regression analysis of risk factors for seroprevalence of CBPP.

Variable		*p* value	OR (95% CI)
Age	Young (6-2yrs)	*∗*	*∗*
	Adult (> 2yrs)	0.554	1.3(0.525-3.329)

Sex	Male	*∗*	*∗*
	Female	0.536	1.3(0.598-2.692)

Body condition	Good	*∗*	*∗*
	Poor	0.115	1.8(0.863-3.868)

Season	Rainy	*∗*	*∗*
	Dry	0.018	2.5(1.171-5.415)

Agroecology	Highland	*∗*	*∗*
	Midland	0.534	1.6(0.371-6.768)
	Lowland	0.01	8.4(1.171-5.415)

*∗* means reference group.

**Table 4:** Multivariable logistic regression analysis of risk factors for seroprevalence of CBPP.

Variable		*p* value	OR (95% CI)
Body condition	Good	*∗*	*∗*
	Poor	0.252	1.6(0.722-3.459)

Season	Rainy	*∗*	*∗*
	Dry	.434	1.4(0.608-3.187

Agroecology	Highland	*∗*	*∗*
	Midland	0.541	1.6(0.367-6.752)
	Lowland	0.02	7.3(2.048-25.752)

*∗* means reference group.
